# Aggregation-Induced Emission in Electrochemiluminescence: Advances and Perspectives

**DOI:** 10.1007/s41061-021-00343-9

**Published:** 2021-06-19

**Authors:** Guillermo Moreno-Alcántar, Alessandro Aliprandi, Luisa De Cola

**Affiliations:** 1 Institut de Science Et D’Ingénierie Supramoléculaires (ISIS), University of Strasbourg & CNRS, 8 allée Gaspard Monge, 67083 Strasbourg, France; 2 Institute for Nanotechnology (INT), Karlsruhe Institute of Technology, Hermann-von-Helmholtz-Platz 1, 76344 Eggenstein-Leopoldshafen, Germany; 3 Dipartimento Di Scienze Farmaceutiche, DISFARM, and Istituto Di Ricerche Farmacologiche Mario Negri, IRCCS, University of Milan, Milan, Italy

**Keywords:** Aggregation-induced electrochemiluminescence, Sensing, Electrochemistry, Photophysics, Enhanced emission

## Abstract

**Abstract:**

The discovery of aggregation-induced electrochemiluminescence (AIECL) in 2017 opened new research paths in the quest for novel, more efficient emitters and platforms for biological and environmental sensing applications. The great abundance of fluorophores presenting aggregation-induced emission in aqueous media renders AIECL a potentially powerful tool for future diagnostics. In the short time following this discovery, many scientists have found the phenomenon interesting, with research findings contributing to advances in the comprehension of the processes involved and in attempts to design new sensing platforms. Herein, we explore these advances and reflect on the future directions to take for the development of sensing devices based on AIECL.

**Graphic abstract:**

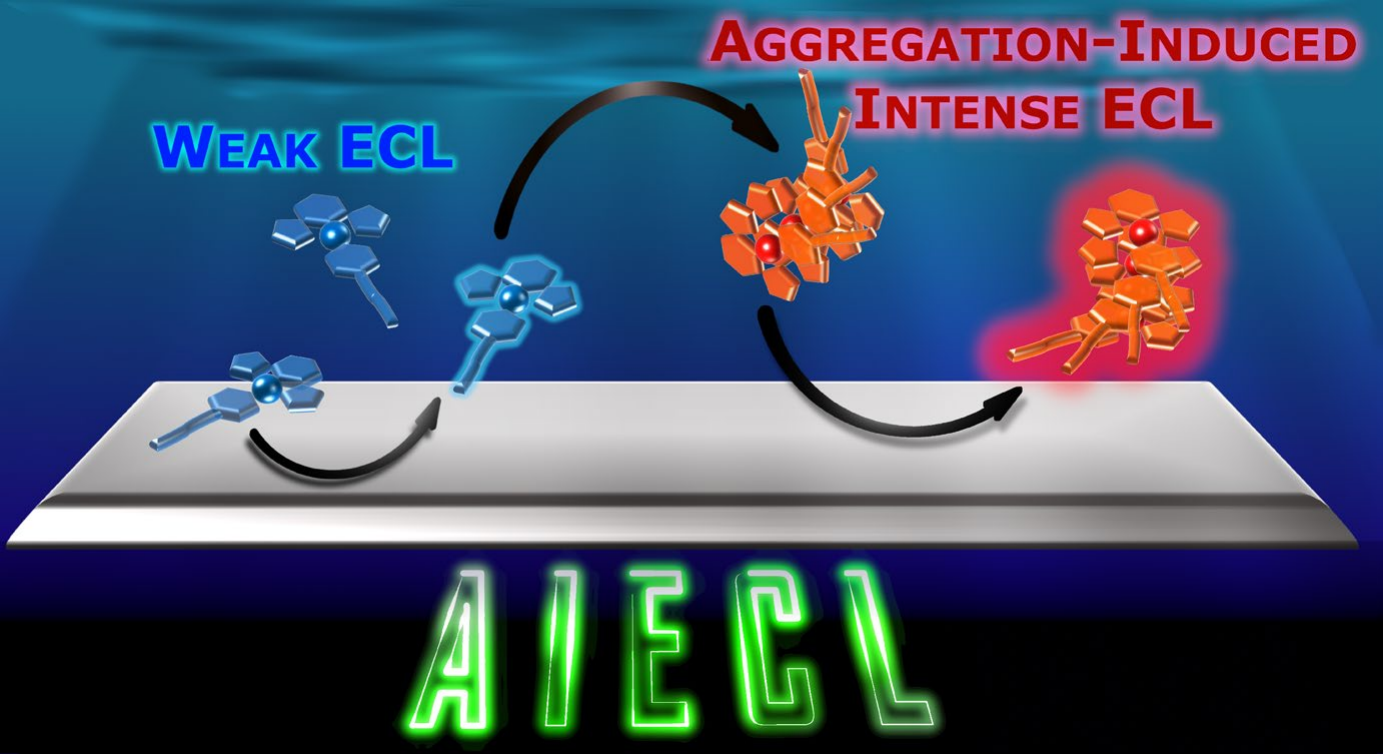

## Aggregation-Induced Electrochemiluminescence

**Fig. 1:**
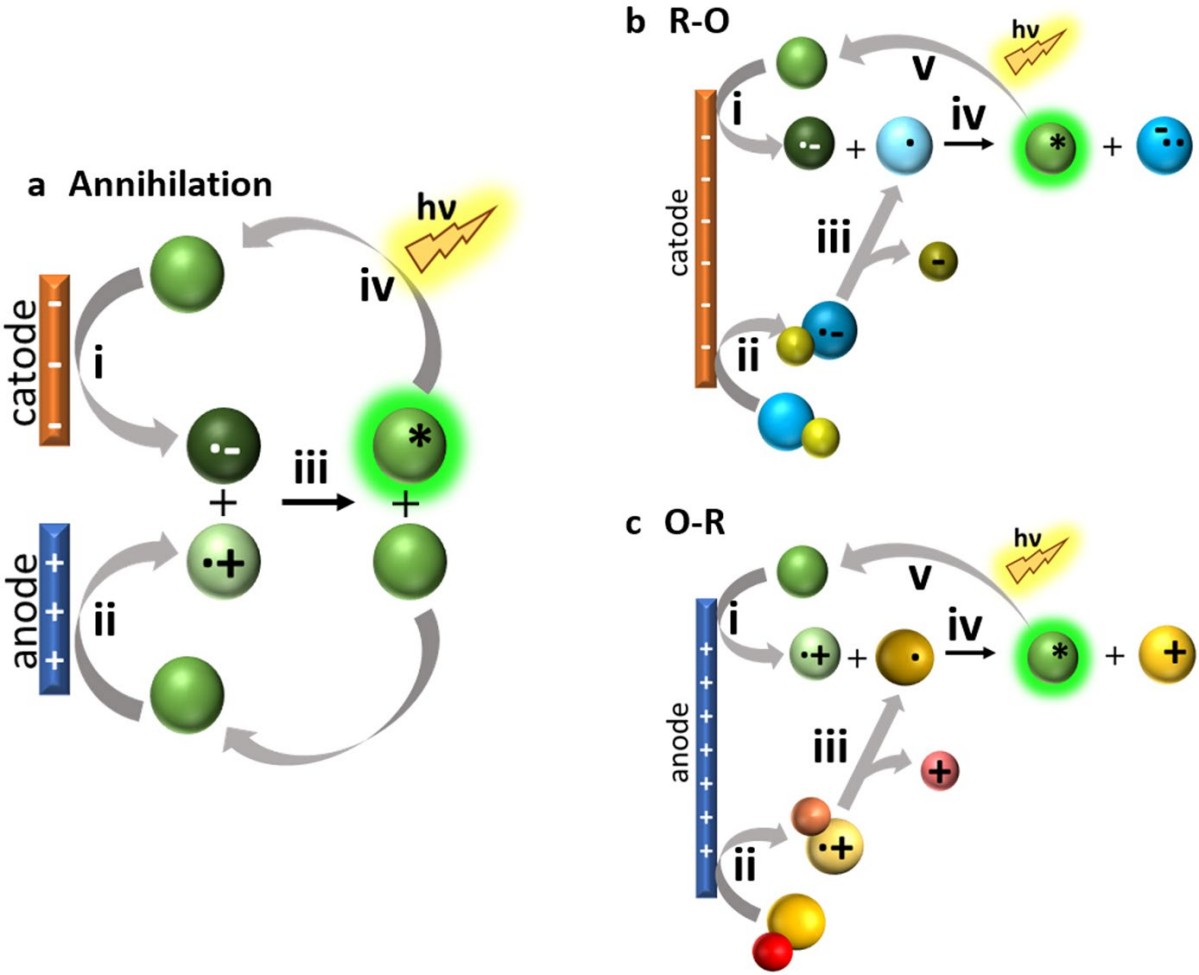
Electrochemiluminescence (ECL) mechanisms. **a** Annihilation: first the reduction (*i*) and oxidation (*ii*) of the luminophore take place; then the annihilation reaction between the oxidized and reduced luminophore radicals (*iii*) produces the electronically excited form of the luminophore that relaxes to the ground state upon light emission (*iv*). **b** Co-reactant reduction–oxidation (*R–O*): the reductions of both luminophore (*i*) and co-reactant (*ii*) take place; then, after a subsequent reaction (generally bond cleavage), the reduced form of the co-reactant generates a strong oxidizing radical (*iii*) which exergonically reacts with the reduced luminophore (*iv*) to generate an excited state, which in turn relaxes to the ground state upon light emission (*v*). **c** Co-reactant oxidation–reduction (*O–R*): the oxidations of both luminophore (*i*) and co-reactant (*ii*) take place; then the co-reactant decomposes to form a strong reducing radical (*iii*) that subsequently reduces the luminogen (*iv*) to generate the electronic excited state that relaxes to the ground state upon light emission (*v*)

Electrochemiluminescence (ECL) is a technique that uses an applied electrical bias to generate reactive species in the vicinity of an electrode that afterwards undergo subsequent electron-transfer reactions, leading to the formation of a luminescent excited state [1]. Although the discovery of this phenomenon was reported at the beginning of the twentieth century [2, 3], the first acknowledgeable studies were performed by Hercules [4], Visco and Chandross [5], and Santhanam and Bard [6] in the 1960s. Since then, and particularly in recent decades, the understanding of the processes and the applications of ECL have rapidly expanded [7]. In ECL, light emission can be obtained mainly by two mechanisms (see Fig. 1). If the reaction takes place between the oxidized and the reduced forms of the luminophore, both generated sequentially at the working electrode, the mechanism is defined as annihilation. In contrast, if the reaction involves the use of sacrificial reagents that upon oxidation/reduction decompose into highly energetic species able to react with the luminophore, thereby generating the excited state, during a single potential step or scan, the ECL mechanism is denoted as the co-reactant type. As opposed to chemiluminescent systems where all reagents are irreversibly consumed, in ECL, the luminophore is regenerated after each cycle; consequently, a large number of photons can be produced. Such a signal generation mechanism combined with the great spatial and temporal control derived from the electrochemical generation of reactive species are the strengths of ECL compared to other analytical techniques. Moreover, the absence of background noise, since no light for excitation is needed as in traditional photoluminescent (PL) measurements, results in an extremely high signal-to-noise ratio and high selectivity [8]. Therefore, it is not surprising that nowadays, ECL is used widely as a clinical diagnosis technique and has even become the gold standard in hospitals all over the world [9]. In addition, its applications are not limited to the biomedical field but also for food and water analysis [10, 11] as well as explosives detection [12, 13], just to cite some of the technique’s many applications.

Since the luminophore is regenerated at each cycle the emission intensity is not only dictated by the intrinsic photoluminescent properties of the compound but also by the rate of the redox reactions responsible for the formation of the excited state once the voltage is applied. Therefore, an efficient ECL luminophore must possess suitable redox properties, be stable in its oxidized or reduced state and possibly have excellent PL properties. For diagnostics and biomedical applications, the luminophore must also possess a good degree of solubility in aqueous media where biological analyses are usually performed. Keeping into account also the role of the spin statistics in the formation of the exciton [14], the use of phosphorescent emitters have been found to outperform fluorescent dyes, resulting in the field being dominated by transition metal complexes, in particular ruthenium and iridium complexes [15–21]. Unfortunately, phosphorescent compounds possess relatively low photoluminescence quantum yield (PLQY), especially in aerated conditions as elemental oxygen is an effective quencher of the long-lived excited triplet state. Even for the fluorescent probes, water and polar solvents are often not ideal media for the emission properties, compared with an organic apolar solvent [22]. An aqueous solution can detrimentally affect the emissive behavior by acting as an electron or proton acceptor through hydrogen bond formation [23] as well as by promoting the formation of excimers or exciplex [24]. Such limitations have motivated researchers to exploit new approaches to increase the performance of luminophores, such as by employing nanomaterials [25–27] and supramolecular aggregates [28, 29]. The aggregation-induced emission (AIE) [30–34] and aggregation-induced emission enhancement (AIEE) [35–37] phenomena could be considered to be an ideal tool to overcome the limitation exerted by the media. AIE systems require a careful molecular design in order to make them readily aggregate in solution but introducing steric hindrance renders them capable of avoiding the establishment of detrimental π–π stacking interactions that typically lead to a quenching of the emission. Upon the aggregation of certain systems, a rigidification of the structure takes place that blocks the non-radiative pathways due to rotation and vibration of the moieties forming the fluorophore and favoring the radiative transition. Furthermore, the molecular packing can shield the excited state from the media, preventing unwanted bimolecular interactions, such as with dioxygen, that would lead to emission quenching.

**Fig. 2:**
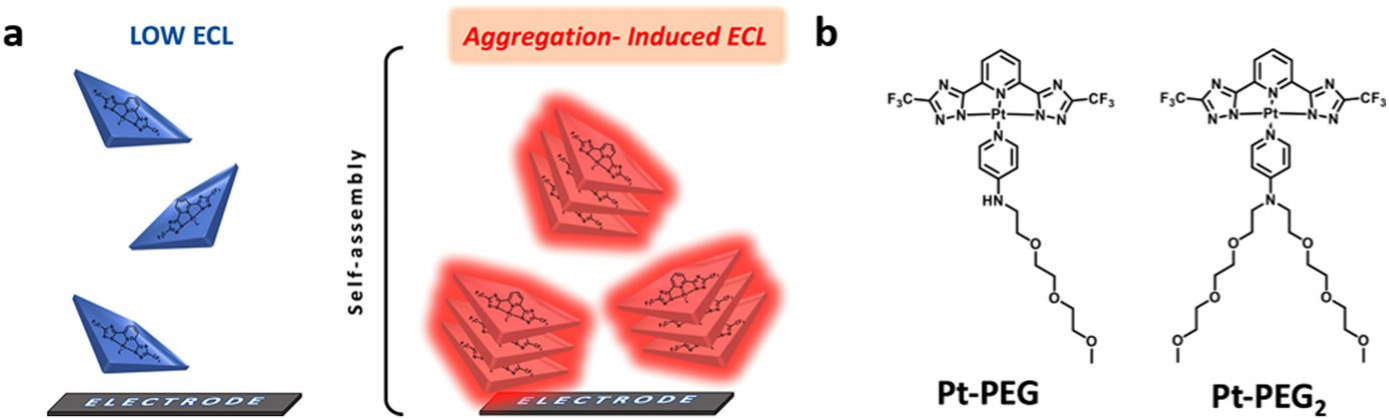
**a** Aggregation-induced electrochemiluminescence (AIECL) observed upon aggregation of neutral Pt(II) amphiphilic compounds. **b** Structures of the Pt(II) amphiphilic complexes **Pt–PEG** and **Pt–PEG2** displaying AIECL. Adapted with permission from Carrara et al. [38]. Copyright 2017 American Chemical Society

The use of AIE for ECL was described for the first time by De Cola et al. in 2017 [38]. In contrast to standard AIE luminophores, in which the structure is designed to prevent electronic interactions upon aggregation, the authors took advantage of the tendency of Pt(II) complexes to establish metallophilic interactions to change not only the optical properties of the compounds but also the redox potentials. Indeed, as shown in Fig. 2a, when Pt(II) complexes are close enough to each other (< 3.5 Å), the dz^2^-orbitals can interact, leading to the formation of new molecular orbitals that can in turn lead to electronic transitions different in nature from those of the monomeric species. In particular, the lowest excited state for the aggregated Pt(II)complexes containing conjugated ligands has a metal–metal–to–ligand charge transfer (MMLCT) character. This results in a bathochromic shift of the emission, often with an enhancement of the PLQY. Moreover, this d–d interaction causes a decrease in the oxidation potential of the complex since the highest occupied molecular orbital (HOMO) is destabilized. The shift towards lower energy favors not only the detection of the emitted light, being in the visible and even in the near-infrared (NIR) region, but also allows the oxidation of the Pt(II) units, which can occur at a potential that can be reached by the common co-reactants or by applying a low voltage at the electrode. Therefore, ECL that is not possible with monomeric species becomes available upon their self-assembly in discrete structures in which electronic interactions are present. This is the reason why this new phenomenon has been defined as aggregation-induced electrochemiluminescence (AIECL), i.e. to stress that the aggregation leads to ECL-active materials. As shown in Fig. 2, the investigated Pt(II) amphiphilic complexes consist of a tridentate chromophoric ligand and an ancillary pyridine functionalized with one (**Pt-PEG**) or two (**Pt-PEG**_**2**_) hydrophilic triethylene glycol chains. The overall charge has been kept neutral to promote aggregation in polar media while the hydrophilic chains have been introduced to promote their solubility in water. While **Pt-PEG** is almost insoluble in water, **Pt-PEG**_**2**_ is able to form a stable orange colloidal dispersion in pure aqueous solutions and its ECL performance outperforms the efficiency of the classical ECL reference [Ru(bpy)_3_]^2+^[7, 16, 39].

## Current Advances and Applications

Since the first report of AIECL, several papers have been published that have expanded and diversified the library of luminophores suitable for this technique, ranging from small molecules, both organic [40–42] and inorganic [43], to polymeric arrangements [44] and composites [45]. Diverse types of aggregates, such as nanoparticles (NPs), nanocrystals (NCs) [47], polymeric dots (Pdots) [44] and many others, have been shown to display AIECL. In all these cases, the mechanisms of triggering the ECL upon aggregation remain the same: changing the redox properties, introducing new excited states or changing the nature of the emissive state—and, of course, rigidification of the systems. The availability of a larger library of luminophores has incentivized the application of AIECL in analytical assays to detect diverse analytes of interest, ranging from heavy metals to biologically relevant molecules [46, 48]. AIECL has proven to be a way to extend ECL methodologies to water by harvesting the tendency of hydrophobic luminogens to aggregate in aqueous solution and has already shown superior efficacies and selectivity. Avoiding the more common aggregation-caused quenching (ACQ) is a challenge in the design of systems, but the number of new creative approaches is increasing. Regarding the scope of this review, we consider AIECL processes to be those for which the ECL measurements are performed on aggregates formed in solution, frequently by the addition of non-solvents, as well as observations of the formation and deposition of aggregates of a defined shape and size on the electrode surface prior to the ECL measurements. Some of the more remarkable results are discussed in later sections of this article.

### Small Molecule-Based AIECL

**Fig. 3:**
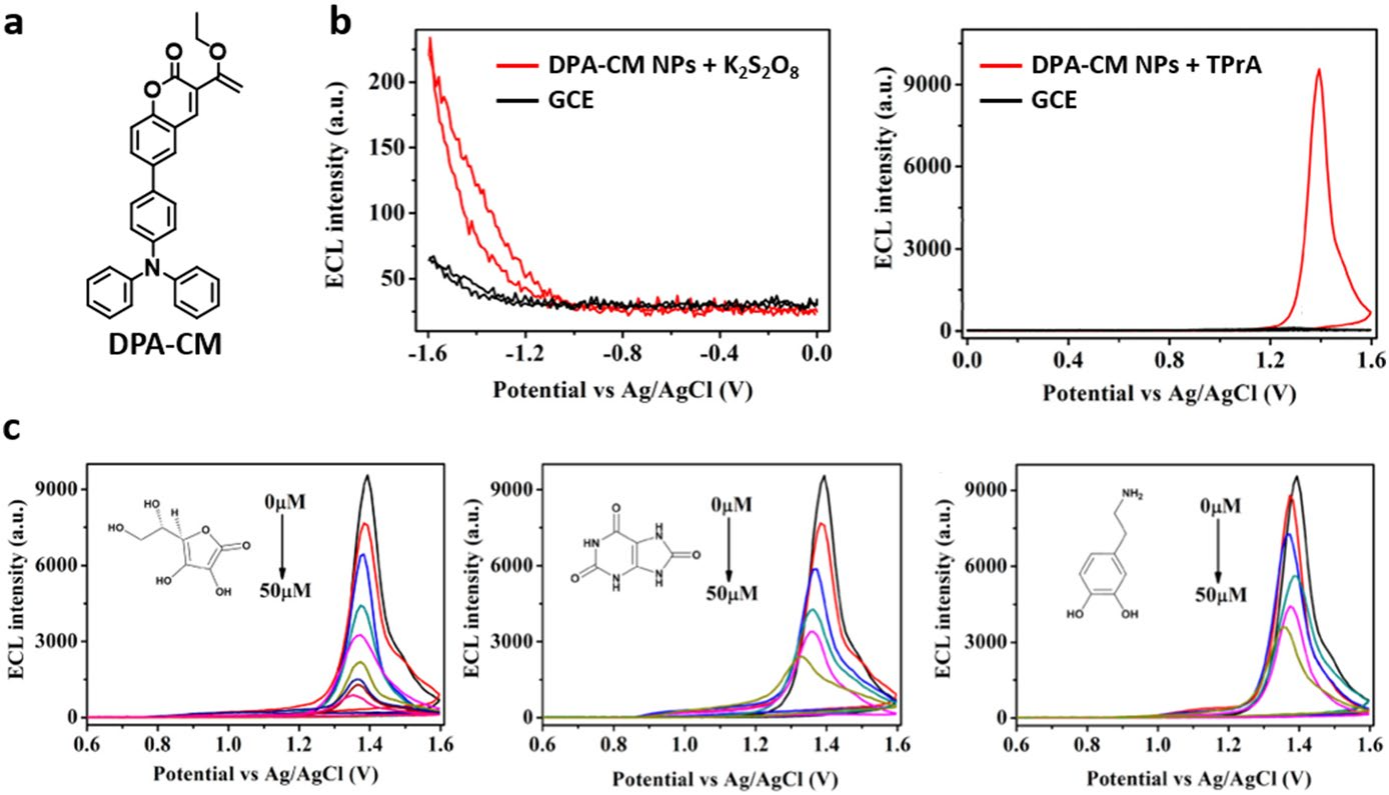
**a** **DPA–CM** (6-[4-(*N*,*N*-diphenylamino)phenyl]-3-ethoxycarbonyl coumarin) structure. **b** ECL intensity potential observed in the presence of in 0.1 M phophate buffered saline (PBS; pH 7.40) containing 50 mM K_2_S_2_O_8_ or 50 mM tripropylamine (*TPrA*). Scan rate: 0.1 V/s. for bare glassy carbon electrodes (*GCE*; black trace) and **DPA–CM** nanoparticles (**DPA–CM NPs**)-modified GCE (red trace).** c** ECL intensity potential of the **DPA–CM NPs**-modified GCE in 0.1 M PBS (pH 7.40) containing 50 mM TPrA and different concentrations (0, 0.05, 0.1, 0.5, 1.0, 5.0, 10, 25, 50 μM) of acetic acid (left), uric acid (middle) and dopamine (right). Adapted with permission from Liu et al. [46]. Copyright 2017 American Chemical Society

Shortly after the description of our AIECL system [38], Zhang and collaborators reported the first AIECL active organic NPs [46]. Their design, based on a donor–acceptor molecule, namely 6-[4-(*N*,*N*-diphenylamino)phenyl]-3-ethoxycarbonyl coumarin (**DPA-CM**; Fig. 3a), NPs of 5.8 nm were formed by a reprecipitation method and deposited on glassy carbon electrodes (GCE). The **DPA-CM NPs** exhibit AIECL in the presence of K_2_S_2_O_8_ or tripropylamine (TPrA). As the latter is far more intense, the oxidation–reduction mechanism was proposed as the most favorable (Fig. 3b). Further, the AIECL signal quenching by different biologically relevant analytes (ascorbic acid, uric acid, and dopamine) was shown to be applicable for the detection of these analytes in the 0.05- to 50-μM linear interval, thereby also representing the first application of AIECL in sensing (Fig. 3c).

AIE luminogens result a straightforward choice in the quest for AIECL luminophores. To date, most of the existing systems proving electrochemically generated luminescence upon aggregation are based on well-known aggregation-induced emitters. Tetraphenylethylene (TPE) is among the most explored AIE luminophores. In the molecular form, TPE and its derivatives present a highly dynamic propeller motion; as a result, they are not—or poorly—emissive when dissolved in solvents. In contrast, TPE aggregation impairs free rotation of the phenyl groups and thus deactivates non-radiative relaxation pathways, promoting an enhanced emission [40–42]. AIECL-based on TPE derivatives is typically seen as an oxidation–reduction process, in which the motion restriction plays a key role, in agreement to what has been observed for photoluminescence.

**Fig. 4:**
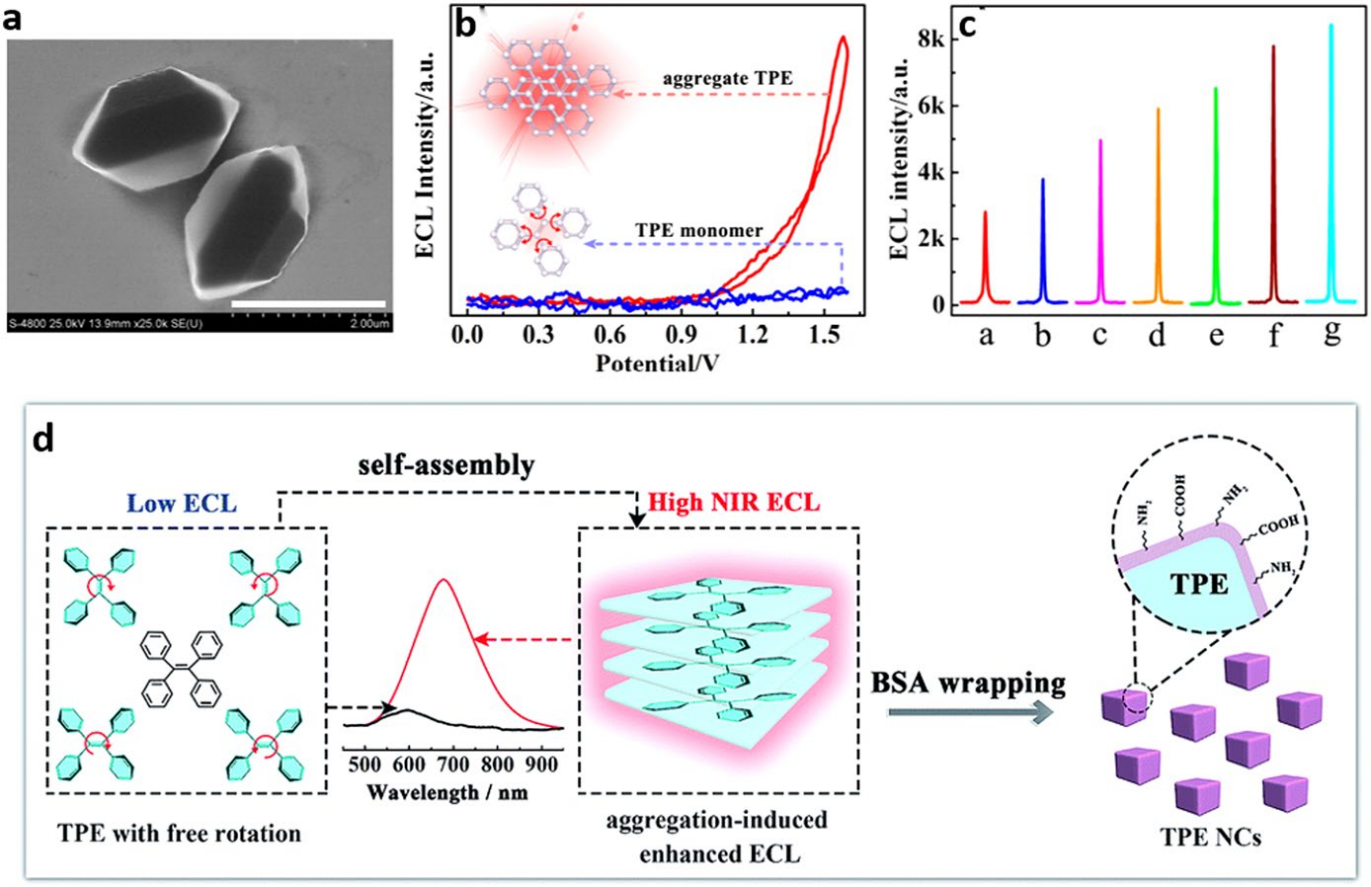
**a** Scanning electron microscopy image of the tetraphenylethylene (*TPE*) microcrystals (scale bar 2 μm). **b** ECL-potential profile for bare GCE in 0.1 M TBAPF_6_ THF solution containing 1 mg mL^−1^ TPE monomers and 20 mM triethylamine (TEA; blue trace), and in 0.1 M PBS containing 1 mg﻿ mL^−^^1^ TPE microcrystals (**TPE MCs**) and 20 mM TEA (red trace). **c** ECL responses of the developed biosensor incubated with different Mucin 1 concentrations:* a* 1 fg﻿ mL^−^^1^* b* 10 fg﻿ mL^−1^* c* 100 fg﻿ mL^−^^1^* d* 1 pg﻿ mL^−^^1^* e* 10 pg﻿ mL^−^^1^,* f* 100 pg﻿ mL^−^^1^,* g* 1 ng mL^−1^. **d** Schematic AIECL NIR-emission observed in **BSA–TPE NCs**. Panels **a**–**c** Adapted with permission from Jiang et al. [49]. Panel **d** Adapted from Liu et al. [47] Article [49], copyright 2019 American Chemical Society. Article [47], with permission of The Royal Society of Chemistry

In this regard, Yuan and collaborators [47, 49, 50] have shown AIECL in TPE microcrystals (**TPE MCs**) formed by surfactant-assisted self-assembly (Fig. 4a). While faint ECL was displayed by the dissolved form of TPE in tetrahydrofuran (THF) (20 mM), the microcrystalline form showed an intense red ECL emission (*λ*_max_ = 675 nm) signal in PBS dispersion using triethylamine (TEA) as co-reactant (Fig. 4b). These MCs have been used for building a biosensor for the detection of a cancer biomarker, Mucin 1 (MUC1), a membrane protein, achieving a linearity range of 10^−3^−10^3^ pg mL^−^^1^ and a limit of detection (LOD) of 0.29 fg mL^−1^ which is comparable to the best reported MUC1 sensing platforms (Fig. 4c) [49]. Further, the same group has addressed the origin of the strongly red-shifted AIECL (*λ*_max_ = 678 nm) in relation to the photoluminescence (*λ*_max_ = 440 nm) of bovine serum albumin (BSA)-coated TPE NCs (**BSA–TPE NCs**). Employing cyclic voltammetry, these authors observed a lowered energetic gap in comparison with that of the PL, suggesting the that the existence of surface states originating the ECL was different from the bulk bandgap observed in the PL; the NIR emission efficiency relative to [Ru(bpy)_3_]^2+^ was found to be 1.35% (Fig. 4d) [47]. These **BSA–TPE NCs** were further used for developing a highly sensitive and selective biosensor for the detection of miRNA-141 [50]

**Fig. 5:**
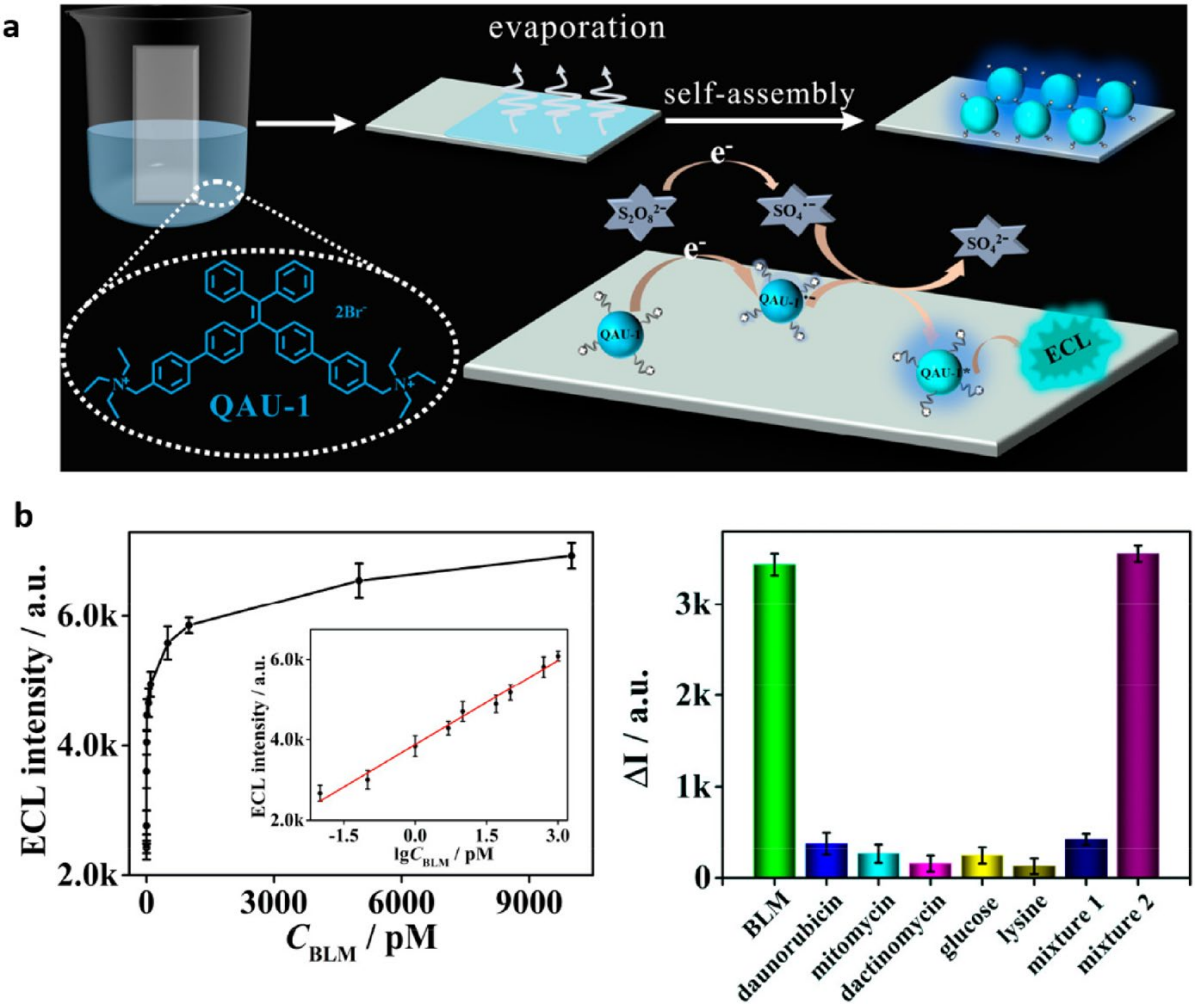
**a** Schematic representation of the indium tin oxide (ITO) glass functionalization with **QAU-1** assemblies and the AIECL mechanism proposed.** b** Left: ECL response of a ferrocene functionalized DNA sequence (Fc-DNA)/**QAU-1**/ITO-based biosensor to the concentration of bleomycin (*BLM*); the inset shows the linearity of the ECL response with the logarithm of the concentration. Right: Change in the ECL intensity of Fc-DNA/**QAU-1**/ITO-based biosensor in the presence of different analytes. Adapted with permission from Lv et al. [52] Copyright 2020 American Chemical Society

Chemical modification of TPE has shown to be a powerful strategy not only for the tuning of the aggregation and emission properties of the materials but, as shown by Lu et al. [51], because it represents a simple way to produce sensing platforms in the aqueous phase. Functionalization of TPE, i.e. introducing positively charged ammonium groups, has been recently explored by the group of Li [52]. The generated positively charged molecule (**QAU-1**) was used to produce self-assemblies with high charge density on the surface of indium tin oxide (ITO) glass which in the presence of K_2_S_2_O_8_ as a co-reactant generates AIECL by a reduction–oxidation (R–O) mechanism, uncommon to other TPE-based luminophores (Fig. 5a). The electrogenerated emission of the assembly was further quenched by electrostatic interaction with a ferrocene functionalized DNA sequence (Fc-DNA) able to recognize selectively the antitumoral bleomycin (BLM). Charge transfer from the TPE aggregates to ferrocene quenches the AIECL. In the presence of BLM, the Fc-DNA is cleaved, turning on the ECL signal; the linear response to BLM allows its detection in a selective manner (Fig. 5b).

Very recently, TPE functionalization with phosphate groups (**TPE-pho**) was exploited by Yan and collaborators to produce an AIECL system capable of switch-off in the presence of alkaline phosphatase (ALP) [53]. While the steric hindrance and the formation of hydrogen bonds within the phosphate groups enhance the rotation impairment, increasing the AIECL signal, the cleavage of the phosphate by the action of ALP group increases the mobility of the TPE moieties, decreasing the response. The system was used as one-step rapid detector of ALP and showed a linear range of 0.1–6.0 U L^−1^ and a LOD of 0.037 U ﻿L^−1^. These authors also analyzed ALP in human serum, reaching recoveries ranging from 95.12 to 113.8% [53].

**Fig. 6:**
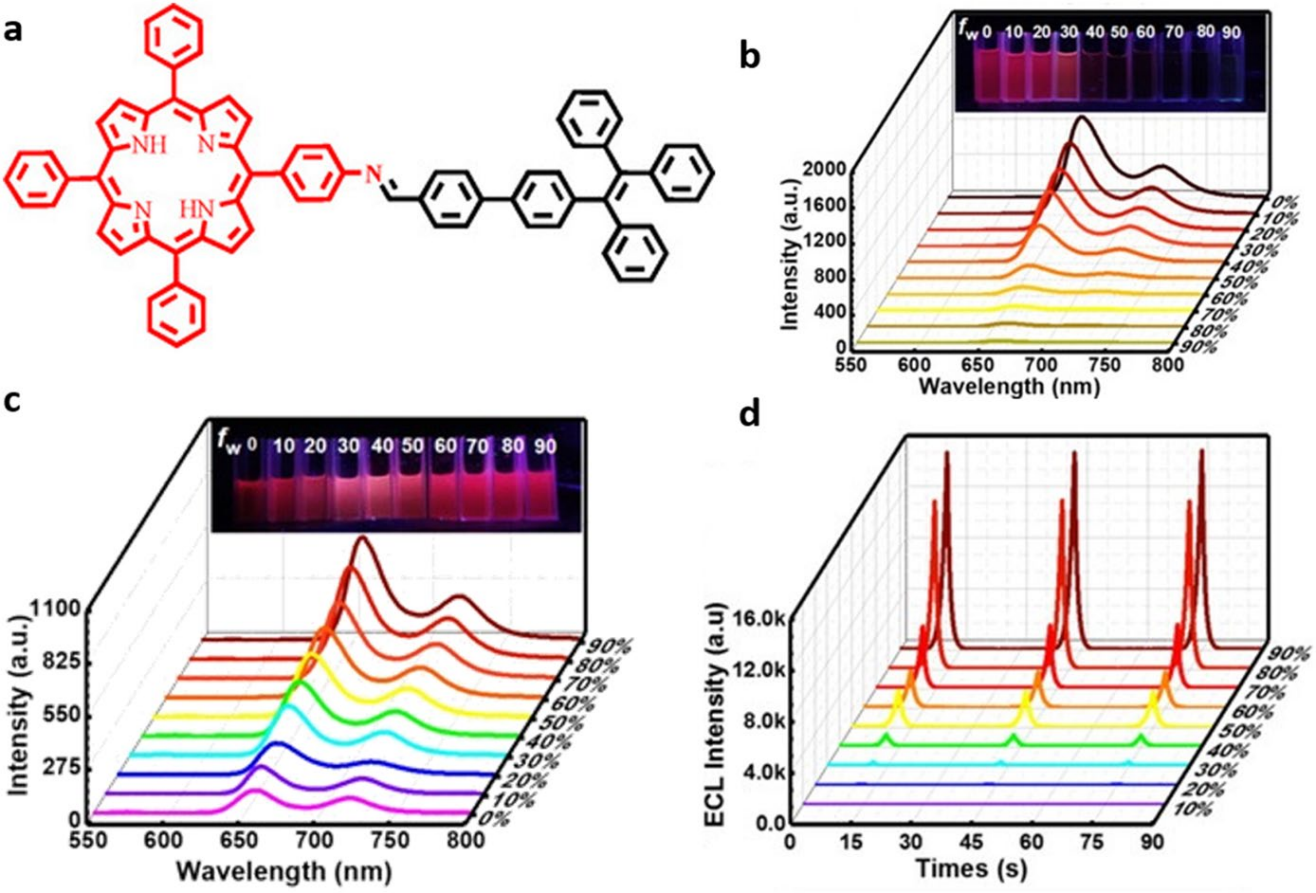
**a** TPE-decorated 5-(4-aminophenyl)-10,15,20-triphenylporphyrin (ATPP). **b** Photoluminescent (PL) aggregation-induced quenching observed upon the increase of water content from 0 to 90% in solutions containing non-functionalized ATPP in DMF. **c** AIE PL emission displayed by** ATPP–TPE** upon increases in water content from 0 to 90%. Insets of **b** and **c** show the corresponding solutions under UV light (365 nm) **d** ECL‐time curves of different degrees of aggregation in** ATPP–TPE**. Conditions: 0.1 M K_2_S_2_O_8_ in 0.1 M PBS (pH 7.5) with 0.1 MKCl. Adapted with permission from An et al. [36]. Copyright 2020 John Wiley and Sons

Another elegant example employing TPE is the approach followed by Lu et al. [54] in which the aromatic moieties were linked to 5-(4-aminophenyl)-10,15,20-triphenylporphyrin (ATPP) (Fig. 6a). Porphyrins, despite their interesting photophysical properties, suffer from a low solubility in water and a high tendency to aggregation due to π-stacking interactions, resulting in quenching of the H-aggregates. Introducing TPE in the ATTP structure, not only changed the character of the aggregates from ACQ to AIE (Fig. 6b, c) but also enhanced the AIECL response of the system through the favorable energy transfer from the TPE moiety to the ATTP. In optimal conditions (90% water content, using K_2_S_2_O_8_ as co-reactant), **ATPP–TPE** reaches a 34% efficiency relative to the benchmark [Ru(bpy)_3_]^2+^ and the intensity signal is stable for more than 20 cycles (Fig. 6d). The chemical functionalization including TPE units for AIECL has also been exploited in systems based on polymers (discussed in following section).

It has been reported that J-aggregates of zinc porphyrin complexes display a strong AIECL [55]. Thus, the choice of methodologies that suppress the formation of the π-stacked H-aggregation and favor the displaced J-aggregates is a strategy that can promote the AIECL in porphyrin-based luminophores. Using surfactant-assisted synthesis, Fu and collaborators [56] obtained the J-aggregates of 5,10,15,20-tetrakis (4-carboxyphenyl)porphyrin (**TCPP**) that by an R–O mechanism, using peroxydisulfate as co-reactant, displays AIECL. This system was applied in the construction of a composed platform for the sensing of Cu^2+^. The authors discovered that the use ofL-cysteine-capped zinc oxide “nanoflowers” (**ZnO@Cys NFs**) improves both the AIECL response and the sensor sensibility through a dual effect: (1) acting as a co-reactant accelerator and (2) acting as an energy donor to populate **TCPP** excited states. Although there was good performance of the sensing platform (linear range 0.001−500 nmol L^−1^ and LOD (0.33 pmol L^−1^), the mechanism of the dispositive emission quenching by Cu^2+^ was not fully addressed. The authors suggest Cu^2+^ strong coordination to the cysteine as the main factor causing the quenching.

**Fig. 7:**
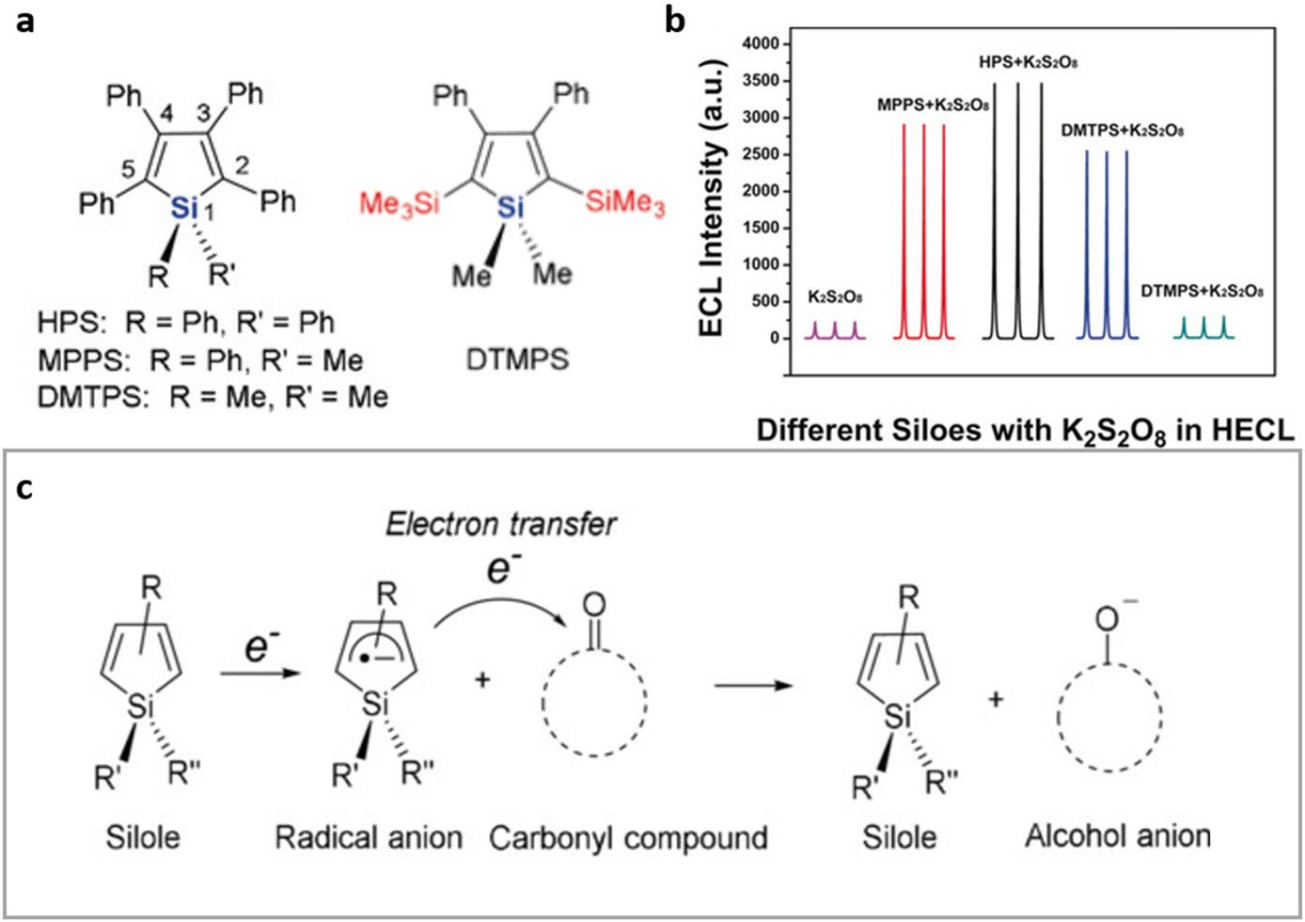
**a** Small AIECL active siloles. **b** ECL intensity of the different siloles in the aggregated state at the electrode surface. Potential window: − 1.6 to 0 V (vs. Ag/Ag^+^); scan rate: 0.1 V s^−1^; 0.1 M PBS containing 0.1 M KCl and 0.1 M K_2_S_2_O_8_, at pH 7.5. **c** Mechanism of the siloles ECL quenching by carbonyl compounds. Adapted with permission from Han et al. [59]. Copyright 2019 John Wiley and Sons

Siloles are another prominent family of AIE active compounds [57, 58] that have recently been explored as AIECL luminogens. Lu et al. [59] demonstrated the potential of 1,1-disubstituted 2,3,4,5-tetraphenylsiloles (Fig. 7a) as AIECL luminophores when deposited on the electrode surface to generate what was denoted heterogeneous aggregation-induced electrochemiluminescence (HAIECL); in solutions of compounds in DMSO with 20% water content weaker ECL was observed, and the behavior in higher water content was not addressed. 1,1,2,3,4,5-Hexaphenylsilole (**HPS**) was found to have the higher HAIECL efficiency amongst the studied compounds, with 37.8% (relative to [Ru(bpy)_3_]^2+^) under optimal conditions, using K_2_S_2_O_8_ as co-reactant (Fig. 7b). Further, the authors proposed the use of **HPS** for the detection of carbonyl compounds, taking advantage of the formation of the silole radical anion intermediary in the ECL mechanism which can react with carbonyls, thus quenching the emission (Fig. 7c). This concept was proved in the sensing of the industrial plasticizer di-*n*-butyl ortho-phthalate (DNBP). More recently, this group has also studied the effects of a 4-substitution on the phenyl groups of tetraphenylbenzosiloles (TPBS), observing the positive effect of electron withdrawing groups on the enhancement of the AIECL response [60]. The competing reduction of Cr_2_O_7_^2−^ was exploited in order to use the system for the detection of hexavalent chromium [60].

**Fig. 8:**
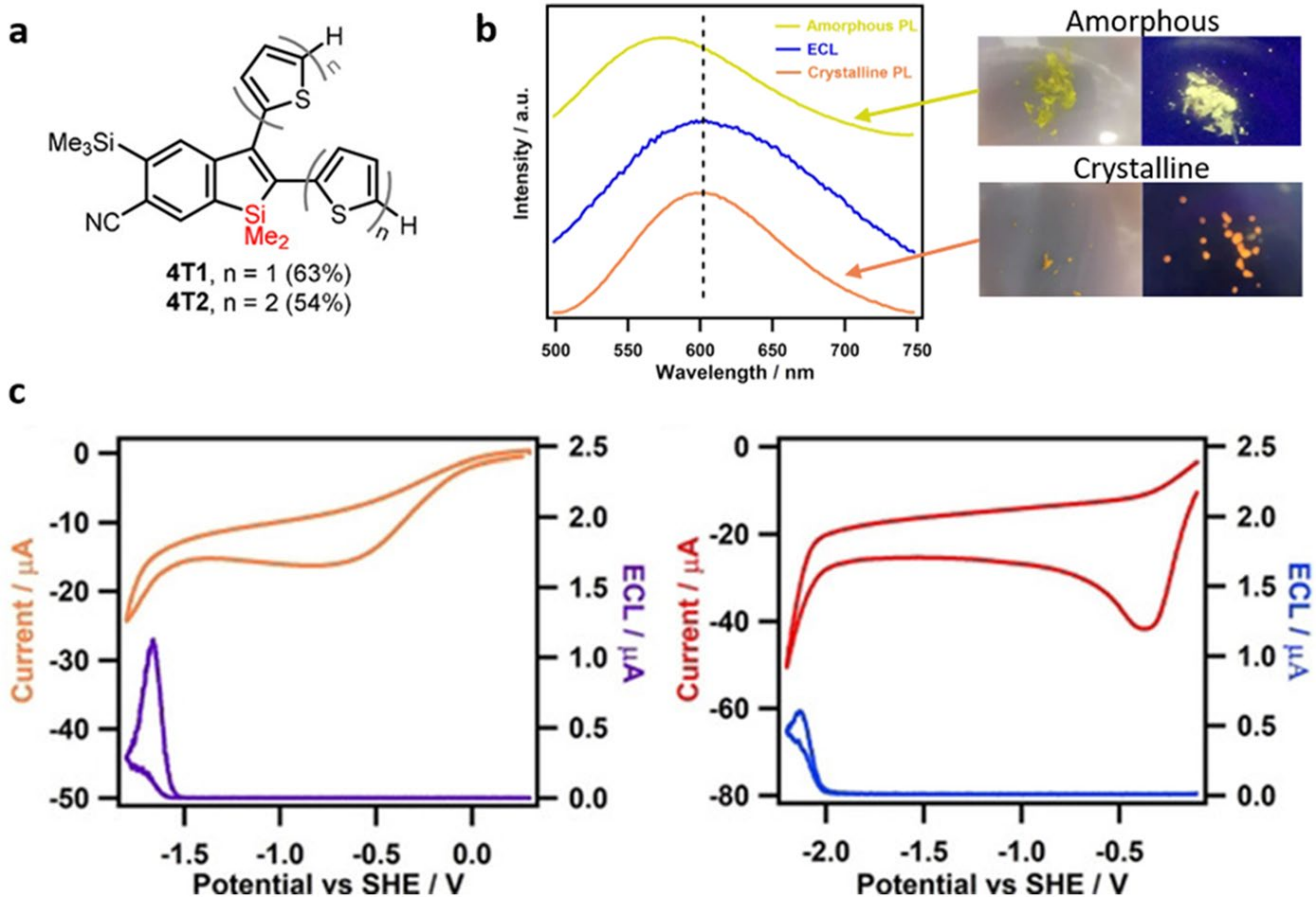
**a** Structure of the benzosiloles **4T1** and **4T2**. **b** Emission spectra of **4T2**, upon photoexcitation, for the amorphous (yellow trace) and crystalline forms (orange trace), and ECL of the films (blue trace) along with photographs of the **4T2** under natural (left) and 365 nm UV (right) light. **c** Cyclic voltammograms and ECL-potential diagrams of **4T2** (left) and **4T1 **(right) films with 5 mM benzoyl peroxide as co-reactant at 0.1  Vs^−1^. Adapted with permission from Yang et al. [61]. Copyright 2020 John Wiley and Sons

An extension of the silole’s work was described by the group of Pagenkopf [61] who extended the concept of crystallization-induced emission enhancement [62] to benzosilole systems displaying enhanced AIECL in the crystalline state rather than in the amorphous aggregates. The synthesized compounds (**4T1** and **4T2**; Fig. 8a) display weak ECL in dichloromethane solutions; in the absence of co-reactants the annihilation mechanism produces ECL signals with an efficiency of 2.3 and 2.1%, respectively, for **4T1** and **4T2** relative to [Ru(bpy)_3_]^2+^ in the same conditions. The addition of benzoyl peroxide (BPO) as a co-reactant reduces the relative efficiency to 0.08 and 0.4%, respectively. Upon the drop-casting formation of crystalline films in GCEs, using a 1:1 water:acetonitrile solvent and upon addition of BPO, as co-reactant, an increase of the efficiency to 2.5% (**4T1**) and 6.5% (**4T2**) has been reported. The mechanism proposed involves a dimerization step, which enhances the emission and causes a red-shift for both solution and amorphous states (Fig. 8b).

One of the first observations of ECL involved 9,10-diphenylanthracene (DPA) as luminophore [6]; recently, DPA cubic NPs (CNPs) have been exploited for the construction of an AIECL immunosensor for the detection of the mycotoxin aflatoxin B_1_ (AFB_1_) [63]. The authors observed an important increase of the ECL signal when the DPA CNPs were deposited on the GCE surface in comparison with the signal observed when the NPs were dispersed in the solution. The mechanistic studies suggested an oxidation–reduction (O–R) mechanism using TPrA as co-reactant. Upon optimization, the constructed immunosensor showed a LOD of 3 fg·mL^−1^ and a linear range of 0.01–100 ng mL^−1^ for the detection of AFB_1_.

**Fig. 9:**
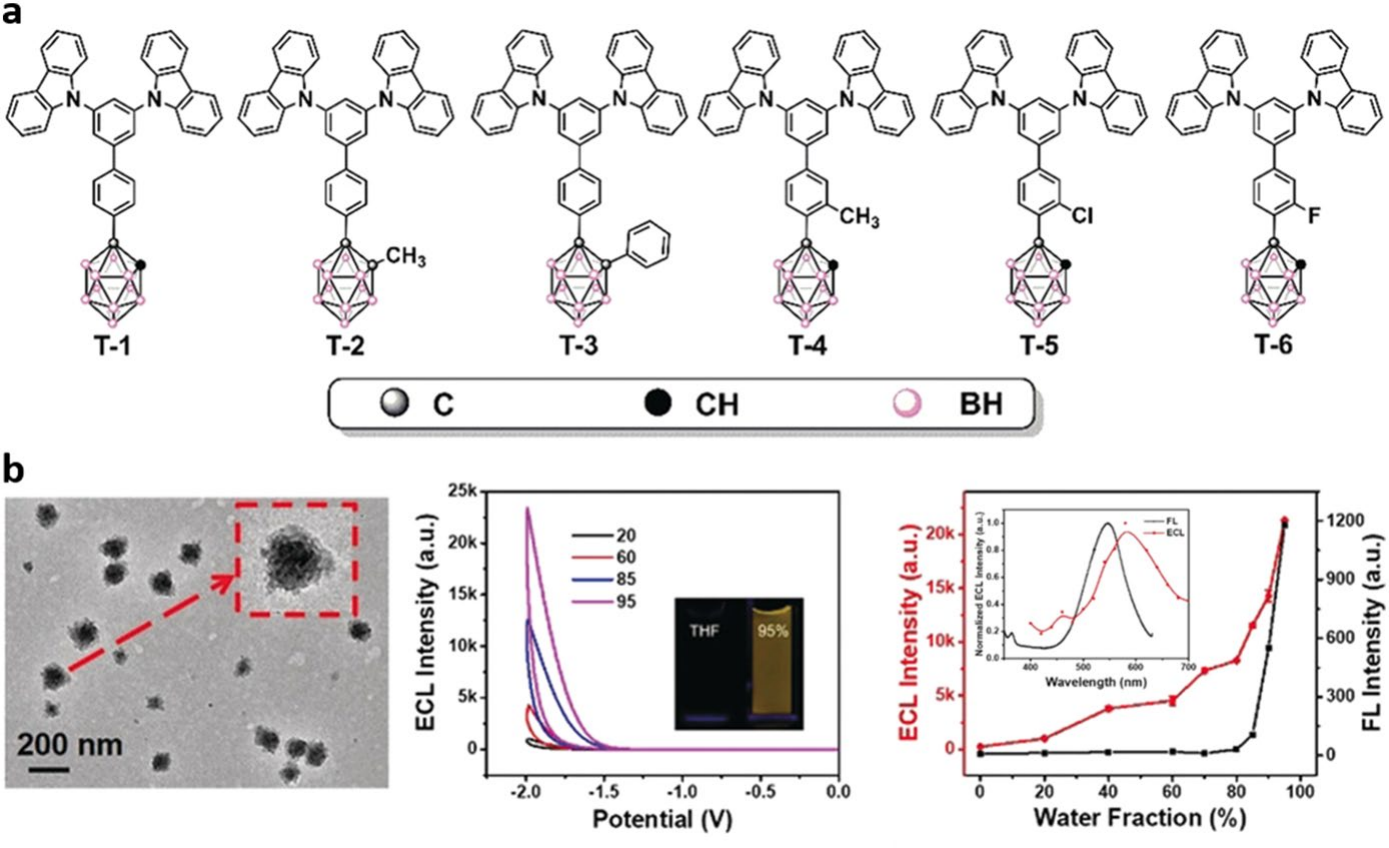
**a** Structures of the carboranyl carbazoles **T-1**—**T-6**. **b** Left: Transmission electron microscopy image of** T-3** aggregates formed in 95% H_2_O. Center: ECL signal enhancement on **T-3** at different water contents; the inset shows the AIE PL. Right: AIECL (red trace) and AIE (black trace) as a function of the water content of the media; the inset shows the AIECL (red) and PL (black) spectra. Adapted with permission from Wei et al. [64]. Copyright 2019 John Wiley and Sons

Wei et al. proposed the use of carboranyl carbazoles as efficient R–O AIECL fluorophores in order to overcome the limited applications of O–R systems in biosensing [64]. In aqueous media, the synthesized molecules (**T-1–T-6**; Fig. 9a) associate in NPs that display AIE behavior, which is remarkable for compound **T-3**. The excellent properties of this compound are thought to be related to the presence of the 1,2-diphenyl-*o*-carboranyl moiety in the structure, which allows a more efficient back-donation. Although all of the reported compounds performed 10- to 40-fold-enhanced ECL emission upon aggregation, **T-3** increased the emission by 221-fold in comparison with its THF solution. It has been observed that smaller particles show a more intense ECL. The red-shifted emission of the ECL peak (582 nm) in comparison with the PL (547 nm) is ascribed to surface state transitions that are less energetic than the bulk transitions (Fig. 9b); in all the cases, K_2_S_2_O_8_ was used as a co-reactant in solutions containing 95% water: 5% THF.

Recently, following up on their interest on the use of donor–acceptor AIE luminophores to generate ECL-active films [65], the group of Liang reported the AIECL behavior of a benzothiadiazole-bistriphenylamine (**BTD–TPA**) [66]. This compound displays an aggregation-induced ECL enhancement of 252-fold when compared with the aggregates formed in water with the compound in THF solution. The authors also produced films in which the use of Au wafers as electrodes boosted the observed signal by a factor of eight compared with the films formed in GCE. This effect was addressed because of the electrocatalytical role of gold in the oxidation processes of both luminogen and coreactant. In addition, the study of the mass amplifying effect of the ECL by the increase of the loading leads to the morphological characterization of the aggregates formed on the electrode, showing that gold acts as nucleating centers that promote the growing of grass-like aggregates not observed in GCE. Under optimal conditions, using triethanolamine (TEOA; 300 mM) as coreactant these grassy aggregates have shown an ECL efficiency of up to 25.6% relative to [Ru(bpy)_3_]^2+^. Finally, the system was used to detect dopamine with a low LOD (3.3 × 10^–16^ M) in a linear range from 10^–15^ to 10^–8^ M [66].

**Fig. 10:**
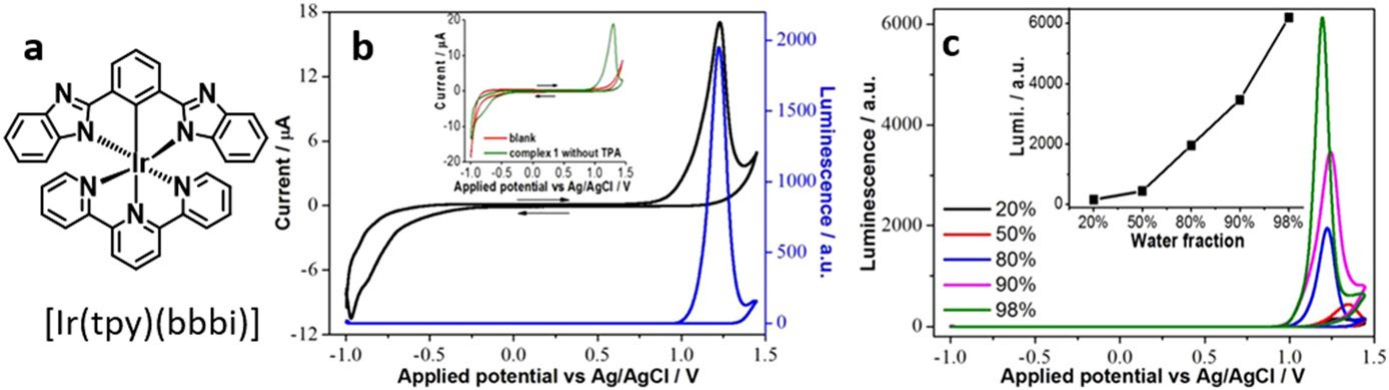
**a** Chemical structure of **[Ir(tpy)(bbbi)]**. **b** Cyclic voltammogram (black trace) and ECL-potential curves of the iridium complex (*c* = 200 μM) in DMSO:H_2_O 20:80; the inset shows the blank curve (red trace). Conditions: 1 mM TPrA and 100 mM NaCl in 10 mM PBS at pH 7.4. Adapted with permission from Gao et al. [43]. Copyright 2018 American Chemical Society

All of the examples illustrated up to this point are for organic systems, even though the first description of AIECL refers to transition-metal coordination compounds. Indeed, metal complexes have also been employed to construct AIECL systems. These include coordination compounds in both polymeric (vide infra) or molecular forms. The group of Ye reported the first molecular iridium complex displaying AIECL [43]. The synthesized **[Ir(tpy)(bbbi)]** (with bbbiH_3_ = 1,3-*bis*(1*H*-benzimidazol-2-yl)benzene) (Fig. 10a) displays weak PL and ECL in the dissolved forms, which the authors attributed to the vibrational and rotational freedom. The restriction of molecular vibrations in the aggregated state activates the radiative paths giving place to enhanced PL and ECL. In 90% water:10% DMSO, **[Ir(tpy)(bbbi)]** forms monodisperse 120-nm aggregates; a further increase of the water content to 98% increases the average size to 160 nm. The increase in the size of the nanoaggregates is consistent with an increase of the ECL signal using TPrA as co-reactant and applying a potential of about + 1.23 V, which the authors attributed to the oxidation of **[Ir(tpy)(bbbi)]** to [Ir(tpy)(bbbi)]^+^ (Fig. 10b). The ECL intensity of these aggregates has shown to be approximately 39-fold more intense than that of its dissolved form and approximately fourfold more intense than that of [Ru(bpy)_3_]^2+^(Fig. 10c). Additionally, the complex aggregates showed a linear increase in ECL intensity upon the addition of bovine serum albumin (BSA), which binds to the surface of the nanoaggregates, diminishing the molecular vibrations; this response suggests the potential of the compound in the development of AIECL biosensors.

**Fig. 11:**
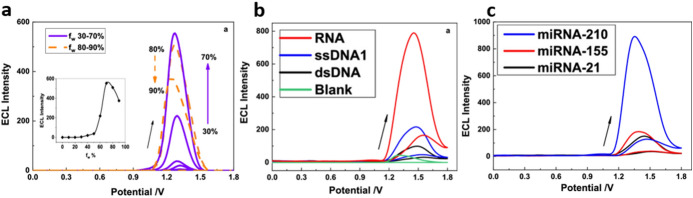
**a** Potential-AIECL signal diagrams of **[Ru**_**2**_**(phen)**_**2**_**Cl**_**2**_**]** (50 μM) at different water contents in CH_3_CN mixtures; the inset shows the change in EICL intensity as a function of the solvent water content. Conditions: 10 mM TPrA, 0.10 M LiClO4 on a GCE, scan rate of 0.10 V/s. **c** ECL of **[Ru**_**2**_**(phen)**_**2**_**Cl**_**2**_**]** aggregates (50 μM) in the presence of 2 μM ssDNA-1, dsDNA or RNA. **c** ECL of Ru(phen)_2_Cl_2_ aggregates (50 μM) in the presence of 2 μM miRNA-210, miRNA-155, and miRNA-2. Conditions for **b** and **c**: 0.10 M LiClO_4_ in 10% (v/v) H_2_O–MeCN using a GCE at a scan rate of 0.10 Vs^−^^1^. Adapted with permission from Gu et al. [67]. Copyright 2020 American Chemical Society

Very recently, Gu et al. reported the use of dichlorobis(1,10-phenanthroline)ruthenium(II) (**[Ru**_**2**_**(phen)**_**2**_**Cl**_**2**_**)]** as AIECL luminophore and its application in the sensing and differentiation of nucleic acids [67]. The PL and AIECL emissions of **[Ru**_**2**_**(phen)**_**2**_**Cl**_**2**_**]** aggregates, using TPrA as co-reactant, were found to be at a maximum in a 70% water:30% CH_3_CN mixture; at higher water content the increase in the size of the aggregates becomes counterproductive, and a decrease in the intensity of the AIECL is observed (Fig. 11a). In 10% H_2_O:90% CH_3_CN, minor AIECL is observed from the pure complex. The addition of different nucleic acid types (i.e. RNA, ssDNA or dsDNA) triggers different aggregation forms that display differentiable ECL signals (Fig. 11b). Furthermore, the ECL response was affected by the abundance of different nucleobases. This approach even allows the differentiation of diverse miRNA strands (Fig. 11c).

A very innovative approach has recently been shown by Wei et al*.* that produced an AIECL fluorophore by confining molecules of *fac*-tris(2-phenylpyridine)iridium(III) (Ir(ppy)_3_) [68]. The aggregation of around 44 molecules within the apoferritin (apoFt) cavity caused a 5.3-fold enhancement of the ECL emission compared to the monomer signal. The encapsulation was achieved by the pH-triggered assembly–disassembly of apoFt. The **Ir(ppy)**_**3**_**@apoF**t bioconjugate was used to construct an immunosensor for the biomarker for squamous cell carcinomas CYFRA21, with a good linearity range (1 pg mL^−1^ – 50 ng mL^−1^) and an interesting LOD (0.43 pg mL^−1^). This type of bioconjugate approach can help to overcome the limited colloidal stability of some strongly insoluble AIECL luminogens [68].

### Macromolecules, Polymeric Structures and Metallic Clusters Matrix-Based AIECL

**Fig. 12:**
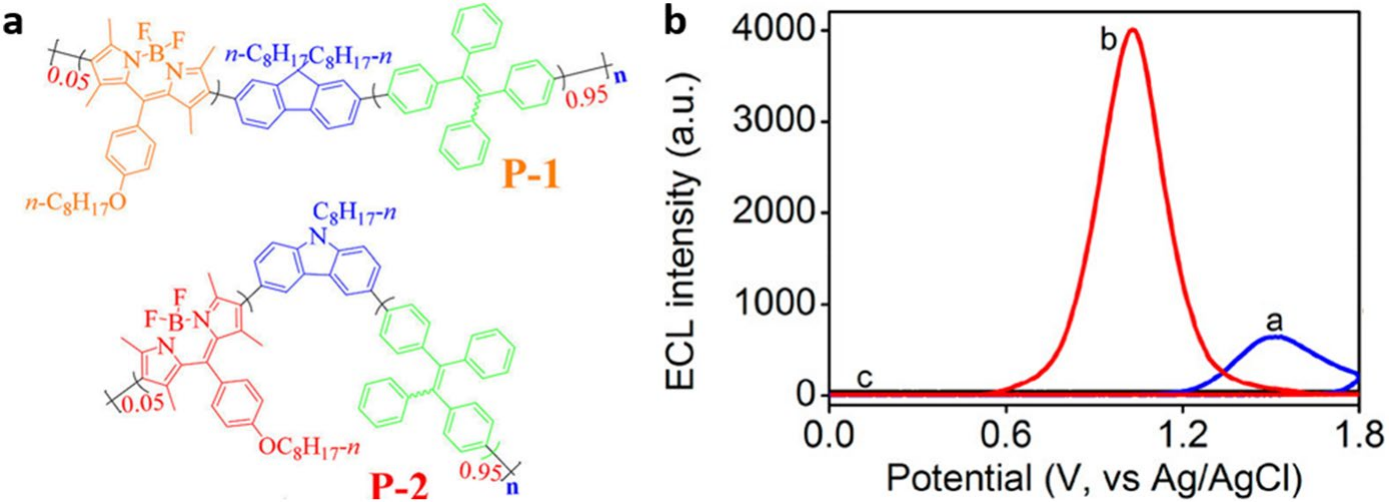
**a** Structures of donor–acceptor-TPE polymers **P-1** and **P-2**. **b** ELC-potential signal of **P-1** Pdots (*a*, blue trace), **P-2** Pdots (*b*, red trace), and GCE (*c*, black trace). Conditions: 0.1 M pH PBS at pH 7.4 in the presence of 0.1 M TPrA as anodic co-reactant. Scan rate: 0.1 V s^−1^. Adapted with permission from Wang et al. [44]. Copyright 2018 American Chemical Society

With the increasing interest in and production of AIECL luminophores and platforms for biosensing, not only small molecules have been used but macromolecular systems have also been explored. An example of such large structures is polymeric dots (Pdots). The robustness, tunability and versatility of Pdots allow the inclusion of active molecular moieties as monomers while preserving their properties. Concomitant to the interest in the aggregation of TPE molecules (see previous section), the TPE moiety has been included in several polymeric designs to generate AIECL. The group of Ju reported donor–acceptor terpolymers including fluorene (**P-1**) or carbazole (**P-2**) donor units, TPE as AIE agents and BODIPYs as acceptor chromophores [44] (Fig. 12a). These polymers were combined with poly(styrene-co-maleic anhydride) (PSMA) to form Pdots. The use of carbazole as a strong donor unit red-shifts the emission of the Pdots, increases the ECL intensity of **P-2** in comparison with **P-1** (by approx. fourfold), and also decreases the anodic peak potential by about 553 mV in the annihilation mechanism. Further, the use of TPrA as co-reactant enhances the ECL signal by promoting an O–R mechanism, and the enhancement is more pronounced for **P-2**, giving an approximately sixfold more intense signal than **P1** (Fig. 12b). **P-1** and **P-2** achieved ECL efficiencies of 5.8 and 11.8%, respectively, when compared with [Ru(bpy)_3_]^2+^. A similar design was used to sense two biologically relevant catechol derivatives: epinephrine and dopamine [69]. The sensing principle relies on the quenching of Pdots emission by the formation of *o*-benzoquinone derivatives as a product of the catechol oxidation. For epinephrine and dopamine, this method has been shown to have linear ranges of 10 to 500 μM and of 10 nM to 100 μM, with detection limits of 3 nM and 7 nM, respectively.

TPE was also recently used to generate conjugated microporous polymers by Suzuki cross-coupling of 1,1,2,2-tetrakis(4-bromophenyl)ethylene (TBPE) with *tris*(4-ethynylphenyl)amine. [70] The system displays an 1.72 % relative ECL efficiency vs. [Ru(bpy)_3_]^2+^ and was applied to sense dopamine using the* o*-benzoquinone-based quenching principle previously described, reaching linearity in the interval of 10 nM to 500 μM with a detection limit of 0.85 nM. The application of TPE-conjugated polymers for sensing has been scaled to a more practical level by the group of Hua et al. [48] who have used boron ketoiminate (BKM)-TPE polymers together with PSMA to build AIECL **BKM–TPE Pdots** of approximately 11 nm in size. Functionalization with DNA opens up the possibility of aggregate selectively with UO_2_^2+^, a common nuclear waste, which is capable of transferring energy to the Pdots, thereby increasing the ECL signal. The detection of UO_2_^2+^ by this method is linear in the range of 0.05–100 nM and presents a very low limit of detection (10.6 pM) for uranyl. These excellent results were translated into a portable device to detect UO_2_^2+^ in natural water sources.

**Fig. 13:**
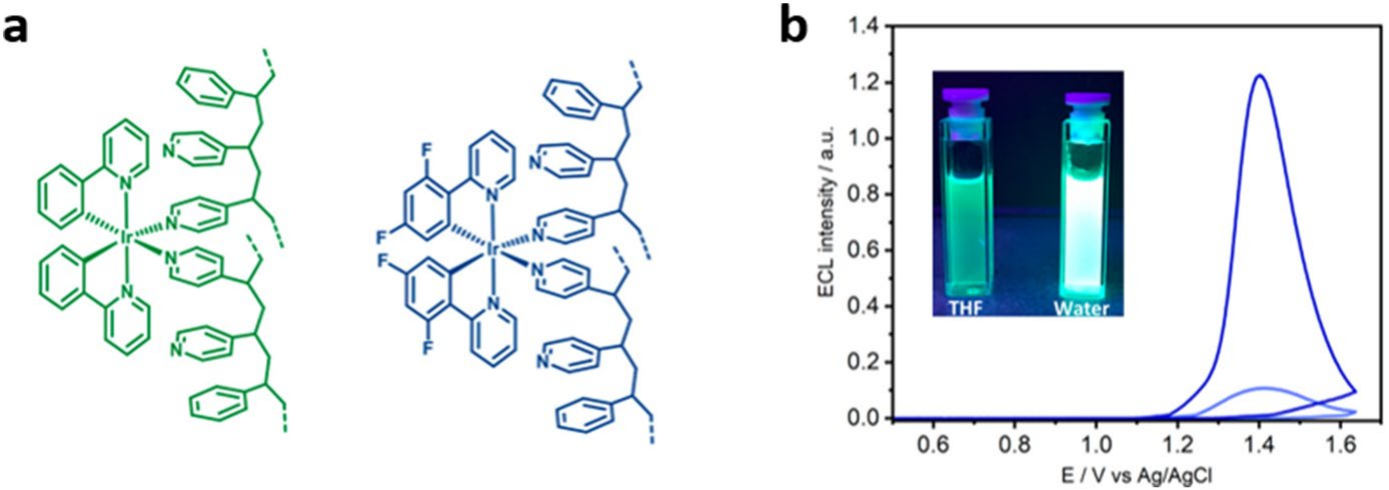
**a** Structure of metallopolymers **[Ir(ppy)**_**2**_**(PVP/S)**_**2**_**]**^**+**^ and **[Ir(dfppy)**_**2**_**(PVP/S)**_**2**_**]**^**+**^. The metal loading was 1 metal:5 monomers and the pyridine:styrene ratio was 2:1. **b** ECL emission-potential diagram of solid-state **[Ir(dfppy)**_**2**_**(PVP/S)**_**2**_**]**^**+**^ polymer (lower intensity: light blue trace) and immobilized polymeric NPs (PNPs) (higher intensity: dark-blue trace) on GCE (scan rate 0.05 V s^–1^ in 0.1 M H_2_SO_4_ water solution using 20 mM Na_2_C_2_O_4_ as co-reactant. Inset shows the PL of **[Ir(dfppy)**_**2**_**(PVP/S)**_**2**_**]**^**+**^ in THF solution and water as PNPs. Adapted with permission from Carrara et al. [71]. Copyright 2018 American Chemical Society

In 2018, Hogan et al. reported AIECL observed in cyclometalated Iridium(III) metallopolymers **[Ir(ppy)**_**2**_**(PVP/S)**_**2**_**]**^**+**^ and **[Ir(dfppy)**_**2**_**(PVP/S)**_**2**_**]**^**+**^ (Fig. 13a) [71]. After addressing the PL and AIE behavior of the compounds by using theoretical methods, the authors showed that the synthesized polymers display ECL emissions in DMF solutions and thin layer films over GCE, following an O–R mechanism in the presence of Na_2_C_2_O_4_ as co-reactant. More importantly, the authors reported the formation of small polymeric NPs (PNPs) of around 44 nm by a reprecipitation method from THF/water (Fig. 13b). The intensity of the ECL signal of **[Ir(dfppy)**_**2**_**(PVP/S)**_**2**_**]**^**+**^ is increased by a factor of 12 when compared to the thin layer signal under similar conditions (Fig. 13c).

The effects of the inclusion of iridium luminophores into polymeric TPE-conjugated matrices attracted the attention of Xu’s group who found that the position of the iridium complexes capping or embedding the polymer is important in the design of these hybrid Pdots [72]. The Ir complex-capped Pdots (**Pdots1**) showed a more intense ECL signal compared to those including different quantities of embedded complex using 2,2′-(butylimino)diethanol as co-reactant. Actually, while the efficiency of the ECL for **Pdots1** (19.8%) was higher than that observed in the non-iridium functionalized Pdots0 (5.05%), the inclusion of an embedded Ir complex into **Pdots2-5** decreased the efficiency (2.92, 1.35, 0.88, 0.150). The gradual decrease in the efficiency from **Pdots2** to **Pdots5** correlates with an increase in the iridium loading of the polymer. This behavior is attributed to the disruption of the TPE conjugation by the inclusion of the Ir-complex units.

**Fig. 14:**
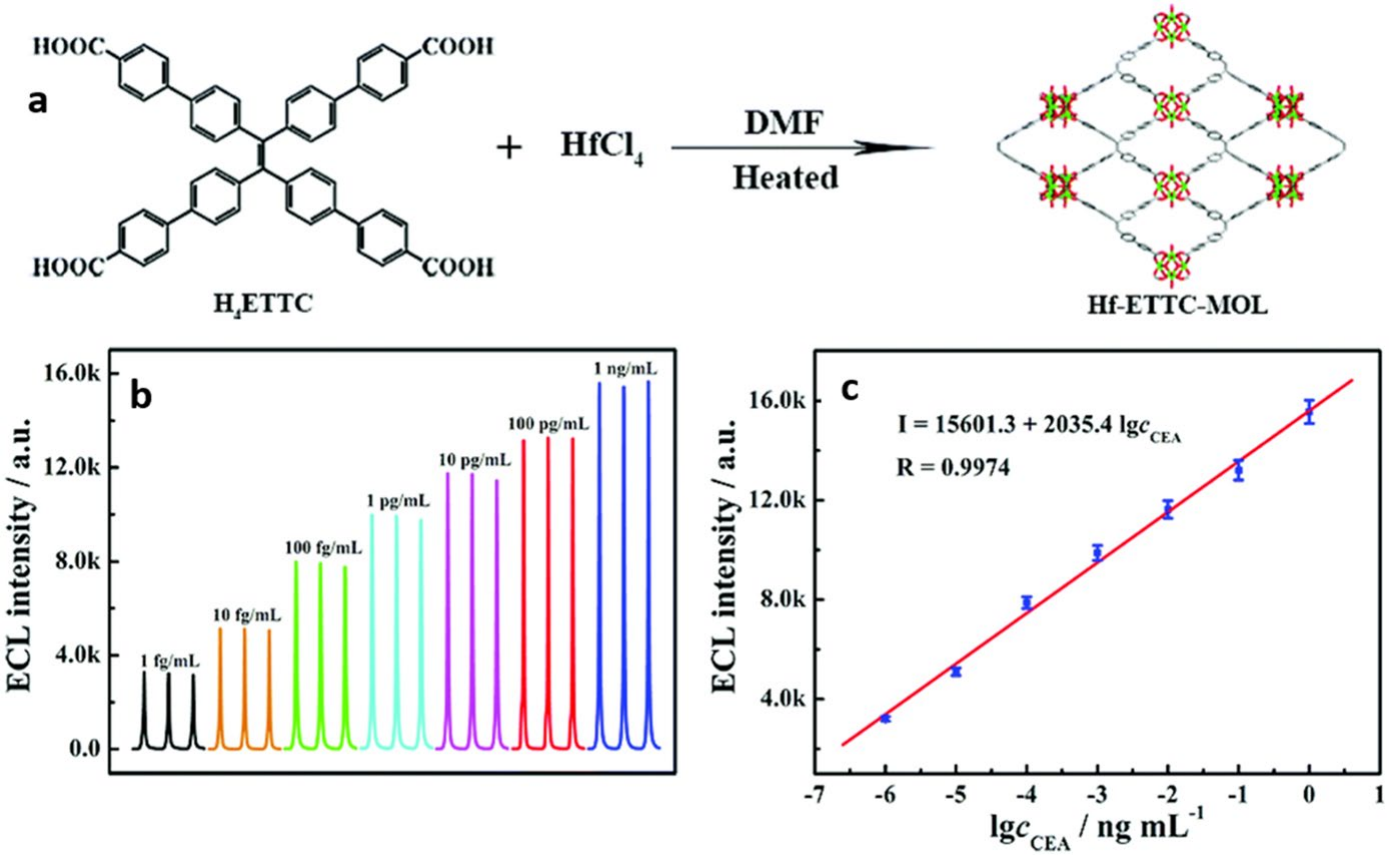
**a** Synthesis of **Hf-ETTC-MOL**. **b** ECL responses of the aptasensor developed using **Hf-ETTC-MOL** when incubated with different concentrations of carcinoembryonic antigen (*c*_*CEA*_). **c** Calibration curve for the ECL intensity vs. the logarithm of c_CEA._. Adapted from Yang et al. [73] with permission from The Royal Society of Chemistry

The construction of two-dimensional polymeric metal–organic layers (MOL) using the TPE-derived ligand **H**_**4**_**ETTC** (4′,4′′′,4′′′′′,4′′′′′′′-(ethene-1,1,2,2-tetrayl)tetrakis {([1,1’-biphenyl]-4-carboxylic acid))} coordinated to hafnium(IV) (**Hf-ETTC-MOL**; Fig. 14a) has shown to have an advantageous ECL performance not only when compared to monomeric or aggregated bare **H**_**4**_**ETTC**, but also against the related three-dimensional metal–organic framework **Hf-ETTC-MOF** [73]. According to Xiao and collaborators, the main factor causing this superior performance of the MOL is the facile diffusion next to the electrode of the entities involved in the O–R ECL process (ions, electrons, co-reactants and intermediaries) through the thin porous layer. This material was then employed in the building of an aptasensor for the detection of carcinoembryonic antigen (CEA), a biomarker overexpressed in several cancer types. The proposed sensor is able to give a linear ECL response in the interval of concentration from 1 fg mL^−1^ to 1 ng mL^−1^ with a LOD of 0.63 fg mL^−1^. This sensor also displayed excellent stability and selectivity for CEA in serum (Fig. 14b, c).

Wang and collaborators created an AIECL system based on adenosine phosphate-coated gold nanoclusters (**AuAXP**; where X stands for the number of phosphate units; M for mono; D for di; T for tri) [74]. In the presence of divalent ions, such as Ca^2+^, Mg^2+^ or Zn^2+^, the **AuAXP** aggregate forms a nano-hydrogel scaffold. This aggregation causes an enhancement in both the PL and ECL response of the material. The ECL response caused by Ca^2+^ has been shown to be modulated by the presence of the Ca^2+^ binding protein calmodulin (CaM). The response demonstrates a linear interval at concentrations ranging from 0.3 to 50 μg mL^−1^, with a LOD of 0.1 μg mL^−1^. This method also allows the assays of CaM in serum samples, with a recovery of 92–117.5%.

The application of EuSe in AIECL was explored by Yafei Li and coworkers [75]. First, they developed a method to control the size of EuSe nanocubes to the range 3 to 70 nm by changing the reaction conditions, in particular the amount of surfactant (1-dodecanethiol). They then observed a regular increase of the ECL signal upon increasing the size of the aggregates and assigned the ECL to a R–O mechanism using K_2_S_2_O_8_ as coreactant. Next, they prepared core–shell structures by covering the EuSe nanocubes successively with CTAB, silica and folic acid; the system was applied as a cytosensor capable of detecting the folate receptor on HeLa cells membrane using a GCE modified with three-dimensional porous graphene-adsorbed Au NPs (3D-GR@Au NPs) [75].

**Fig. 15:**
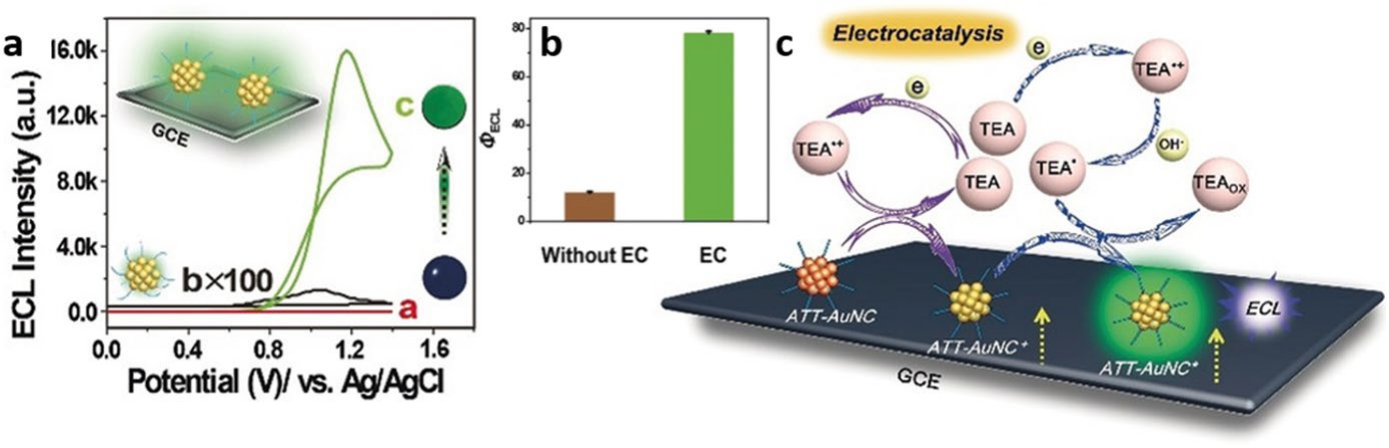
ECL-Potential signals of bare GCE in 0.14 M TEA (red trace), GCE in 0.14 M TEA + **ATT‐AuNCs** (black trace) and **SS‐ATT‐AuNC/GCE** in 0.14 M TEA at 0.2 V s^ − 1.^ Inset: schematic representation of the **SS‐ATT‐AuNC/GCE** and visual ECL of the corresponding electrodes. **b** Effect of the electrocatalysis (*EC*) on the efficiency of **SS‐ATT‐AuNC/GCE** at pH 11.7 in a concentration of TEA = 0.3 M (without EC) or 0.14 M (with EC). **c** Mechanism of the dual electrocatalytic/aggregation enhancement of the ECL signal. Adapted with permission from Liu et al. [76]. Copyright 2020 John Wiley and Sons

An interesting double enhancement effect of ECL was reported by Liu et al. [76]. These researchers discovered that 6-aza-2-thiothymine-coated gold nanoclusters (**ATT-AuNCs**) displayed an enhancement of the ECL upon aggregation by drying the solution on a GCE (**SS‐ATT‐AuNC/GCE**). The solid state ECL of this system displays an important 1200-fold increase compared to the solution ECL (Fig. 15a). Further analysis of the electrochemical behavior revealed that an electrocatalytic (EC) oxidation of the co-reactant TEA improved the oxidation efficiency of the AuNCs. Increasing the TEA concentration avoids its electrocatalytical oxidation due to its concentration-dependent oxidation potential. In a higher TEA concentration, the overall ECL efficiency was reduced from 78% (with EC, c_TEA_ = 0.14 M) to 11.8% (without EC, c_TEA_ = 0.3 M) (Fig. 15b), showing that the dual electrocatalytic/aggregation enhancement of the ECL (Fig. 15c) is a concept that can be further explored for the improvement of other platforms' efficiency.

As summarized herein, diverse approaches towards the generation of different AIECL luminophores, with different performances and possible applications, have already been described. Table 1 summarizes the efficiency of AIECL systems compared to the standard, Ru(bpy)_3_^2+^that have been reported to date. Similarly, the first applications of AIECL for sensing have seen the light with some of these systems, and Table 2 recaps the sensing applications reported so far.

**Table 1:** Reported AIECL efficiencies for the distinct luminophores addressed herein

System	Efficiency (%)^a, b, c^	Co-reactant (c/mM)	Mechanism	Reference
Pt-PEG2	120^b^	TPrA (18)	O–R	[38]
Pt-PEG2	1400^b^	Na_2_C_2_O_4_ (10)	O–R	[38]
TPE-(NO_2_)_4_/K_2_S_2_O_8_	41.2^c^	TPrA (100)	O–R	[51]
ATPP-TPE	34^c^	K_2_S_2_O_8_ (100)	R–O	[54]
HPS	37.8^c^	K_2_S_2_O_8_ (100)	R–O	[59]
4T1	2.5^c^	BPO (5)	R–O	[61]
4T2	6.5^c^	BPO (5)	R–O	[61]
BTD-TPA@Au	26.5	TEOA (300)	O–R	[66]
[Ir(tpy)(bbbi)]	404^b^	TPrA (1)	O–R	[43]
[RuCl_2_(phen)_2_]	100^c^	TPrA (10)	O–R	[67]
P-1	5.8^c^	TPrA (100)	O–R	[44]
P-2	11.8^c^	TPrA (100)	O–R	[44]
TBPE-CMP-1	1.72^c^	TPrA (10)	O–R	[70]
BKM-TPE Pdots	0.5^c^	TPrA (25)	O–R	[48]
BKM-TPE Pdots + UO_2_^2+^	18.6^c^	TPrA (25)	O–R	[48]
Pdots0	5.05^c^	BDEA (25)	O–R	[72]
Pdots1	18.9^c^	BDEA (25)	O–R	[72]
Pdots2	2.92^c^	BDEA (25)	O–R	[72]
Pdots3	1.35^c^	BDEA (25)	O–R	[72]
Pdots4	0.88^c^	BDEA (25)	O–R	[72]
Pdots5	0.15^c^	BDEA (25)	O–R	[72]
H_4_ETTCAgg	10.34^c^	TEA (36)	O–R	[73]
Hf-H_4_ETTC-MOF	15.52^c^	TEA (36)	O–R	[73]
Hf-H_4_ETTC-MOL	27.63^c^	TEA (36)	O–R	[73]
SS-ATT-AuNC	78^c^	TEA (140)	O–R	[76]

^a^Relative to [Ru(bpy)_3_]^2+^/TPrA (100%)

^b^Direct maximum intensity comparison

^c^Integrated intensities quotient

**Table 2:** AIECL-based sensing platforms

System	Analyte	Linear range	LOD	Reference
DPA–CM NPs	Uric acid	0.05–50 μM	0.2 μM	[46]
DPA–CM NPs	Dopamine	0.05–50 μM	0.04 μM	[46]
DPA–CM NPs	Ascorbic Acid	0.05–50 μM	0.04 μM	[46]
TPE MCs	Mucin 1	10^–6^-1 ng mL^−1^	0.29 fg mL^−1^	[49]
BSA–TPE NCs	miRNA-141	10^–7^-1 nM	13.6 aM	[50]
TPE–(NO_2_)_4_/K_2_S_2_O_8_	Iodide	5–2000 nM	0.23 nM	[51]
QAU-1	Bleomycin	0.01–10^4^ pM	4.64 fM	[52]
TPE-pho	ALP	0.1–6.0 U L^−1^	0.037 U mL^−1^	[53]
ZnO@Cys NFs-TCPP	Cu^2+^	0.001–500 nM	0.33 pM	[56]
HPS	DNBP	5–2500 nM	0.15 nM	[59]
BTD-TPA@Au	Dopamine	0.001–10^3^ pM	0.33 fM	[66]
Ir(ppy)_3_@apoFt	CYFRA21	0.001–50 ng mL^−1^	0.43 pg mL^−1^	[68]
P-1	Catechol	2–10^6^ nM	1 nM	[69]
TBPE–CMP-1	Dopamine	10^–6^-1 mM	0.85 nM	[70]
BKM–TPE Pdots	UO_2_^2+^	0.05–100 nM	10.6 pM	[48]
Hf-ETTC-MOL	CEA	10^–6^-1 ng mL^−1^	0.63 fg mL^−1^	[73]
Ca^2+^@AuATP	CaM	0.3–50 μg mL^−1^	0.1 μg mL^−1^	[74]

## Perspectives

It is clear that even in its infancy, the progress made in AIECL during the last 3 years has been brilliant, and interesting applications and promising results have demonstrated the potentialities of this tool. AIECL represents a strategy capable not only of overcoming the limitations of ECL applications in aqueous, biological and environmental media but also to increase the sensing capabilities of existing ECL platforms. The introduction of new or imported concepts that enrich the AIECL toolbox, such as RIM, [49], HAIE-ECL [59], CIEE [61] or SS-AIECL [74], is important to feed the creativity that underlies the conception of more powerful materials capable of detecting the more challenging analytes in the most diverse conditions. Until now, just a handful of the abundant collection of known AIE luminophores have been explored in AIECL, and the expansion to more diverse systems provides AIECL with an important potential that still needs to be explored; in addition, pursuing the de novo design of luminogens specifically for AIECL also has a boundless potential. The interest in AIECL is indeed related to the potential to overcome barriers that typically influence the classical luminophores. The photoexcitation of molecules is regulated by a transition that must satisfy the selection rules of spin and symmetry selection. When the excited state is generated by charge recombination, as in ECL or EL (electroluminescence), such rules can be neglected and the excited state population will be dictated by statistical quantum mechanics consideration, with 25% of the excitons representing singlets and 75% triplets. Therefore, phosphorescent compounds are a better choice for ECL and explain why Ru(bpy)^32+^ has been a standard for decades. If we consider the aggregation of luminophores, this process often leads to quenching because of the formation of low-lying excited states or because of triplet–triplet annhiliation. An accurate choice of emitters can prevent such effects, avoiding π–π interactions or very long-lived species. However, aggregation can be very beneficial for molecules possessing a high number of vibrations or groups that can rotate or that can be quenched by molecules present in solution, such dioxygen. The motions in solution increase the non-radiative decays while the quenchers will both decrease the radiative constants and lower the emission quantum yields. The packing of these molecules in aggregates, where the motion is blocked or strongly hindered, contributes therefore toward increasing the emission quantum yields, reducing the non-radiative pathways but also preventing the diffusion of the quenchers so that they cannot approach the emitters. Finally, for ECL it is very important that the redox properties match the co-reactant’s ability to act as a reducing or oxidizing agent and, therefore, often the oxidation or reduction of the luminophore must be accessible at rather low potentials and possibly reversible. Aggregation can strongly modify such redox behavior since electronic interactions between the molecules will stabilize (or destabilize) the HOMO and/or LUMO (lowest un-occupied molecular orbital) levels.

With such considerations in mind, the design of new luminophores that can undergo AIECL is possible keeping into account that the aggregation process in solution depends on both the concentration of the compound and the media. Indeed, the majority of the reported systems are based on the use of solvent/antisolvent to create the aggregates in the forms of colloidal suspensions; therefore, the final properties are dictated by the preparation protocol. Finally, factors such as morphology, size and shape of aggregates can influence the AIECL performance of the luminophores. Addressing these effects, which are system dependent, is an arduous task required for all the new luminophores and, as discussed above, while an increase in size can in some cases increase the efficiency [75], in others the opposite effect is observed [64]. Likewise, the crystalline and amorphous aggregates can display dissimilar behaviors [62], or even H or J aggregates can exhibit different AIECL response [55]. Also, in very diluted conditions, we expect the compounds to exist in the molecularly dissolved form, or at least in equilibrium with the monomer whose photophysical properties are typically poor. Important analytical applications, such as immunoassays, require the luminogen to be covalently bound to an antibody to form a sandwich complex in a 1:1 ratio. Therefore, the extreme dilution of the luminogen and the steric hindrance exerted by the biological macromolecule would prevent the formation of aggregates and in turn disable the AIECL phenomenon. Therefore, it is of importance to develop new systems that take advantage of the AIE and AIECL phenomena, but which are not affected by media, concentration and preparation protocol. A possible strategy would be to covalently link several luminophores to obtain a discrete system that behaves as an aggregate. Very recently we have shown [77] that by linking together three Pt(II) complexes it is possible to fully benefit from the assembly emerging properties in terms of elongation of the excited state lifetime, protection from the dioxygen quenching and increase in the emission quantum yield (AIE phenomenon). Thanks to the covalent bond, the photophysical properties are not affected by concentration or sample preparations since the “aggregation” is due to intramolecular interactions rather than intermolecular ones. While such an approach has been demonstrated successfully for the case of AIE of neutral Pt(II) complexes, there are no other examples in literature with other luminophores, therefore opening a new avenue in the development of systems with enhanced properties.

## Data Availability

N/A.

## Code Availability

N/A.
